# Outcomes of sonographically-suspected fetal intra-abdominal cysts: Surgical intervention, conservative management and spontaneous regression

**DOI:** 10.3389/fped.2022.1015678

**Published:** 2022-10-18

**Authors:** Shuangshuang Gai, Lixiu Wang, Weizeng Zheng, Bin Xu, Qiong Luo, Jiale Qin

**Affiliations:** ^1^ Department of Ultrasound, The Second Affiliated Hospital, Zhejiang University School of Medicine, Hangzhou, China; ^2^ Department of Ultrasound, Women’s Hospital, Zhejiang University School of Medicine, Hangzhou, China; ^3^ Department of Radiology, Women’s Hospital, Zhejiang University School of Medicine, Hangzhou, China; ^4^ Department of Ultrasound, National Clinical Research Center for Child Health, Children’s Hospital, Zhejiang University School of Medicine, Hangzhou, China; ^5^ Department of Obstetrics, Women’s Hospital, Zhejiang University School of Medicine, Hangzhou, China; ^6^ Key Laboratory of Women’s Reproductive Health of Zhejiang Province, Women’s Hospital, Zhejiang University School of Medicine, Hangzhou, China

**Keywords:** prognosis, ultrasound, abdomen, cysts, congenital abnormalities

## Abstract

**Objective:**

The prenatal diagnosis of fetal intra-abdominal cysts is challenging. This study aimed to evaluate the diagnostic ability of prenatal ultrasound for fetal intra-abdominal cysts and to develop a predictive method for pre- and postnatal outcomes.

**Methods:**

We retrospectively reviewed fetuses with ultrasound-detected intra-abdominal cysts between January 2013 and January 2020. The maternal–fetal clinical characteristics and ultrasound parameters were integrated into a model of pre- or postnatal outcomes.

**Results:**

The study enrolled 190 eligible fetuses, including 94 cases of spontaneous regression, 33 cases of conservative management and 63 cases of surgical intervention. For the 63 cases of surgical intervention, prenatal ultrasound was found to identify fetal intra-abdominal cysts with 80.00% sensitivity (95% CI: 67.03%–89.57%), 37.50% specificity (95% CI: 8.52%–75.51%), 89.80% positive predictive value (95% CI: 83.51%–93.86%), 21.43% negative predictive value (95% CI: 8.80%–43.53%) and 74.60% accuracy (95% CI: 62.06%–84.73%). The predictive model of prenatal spontaneous regression was as follows: y = −3.291 + 0.083 × gestational age + 1.252 × initial diameter, with an area under the curve (AUC) of 0.819 (95% CI: 0.739–0.899) and an optimal cut-off value of 0.74. The large cyst diameter before delivery was an independent predictor of postnatal surgical intervention (*p* < 0.001), with an AUC of 0.710 (95% CI: 0.625–0.794) and an optimal cut-off value of 3.35 cm.

**Conclusion:**

Although ultrasound has a limited ability in the accurate diagnosis of fetal abdominal cysts, a simple method of measuring the diameter can predict fetal outcomes and identify the cases that may require surgical intervention or spontaneous regression.

## Introduction

Fetal intra-abdominal cysts are relatively common, with an incidence of 1 in 1,000 (1, 2). Fetal intra-abdominal cysts may encompass a variety of congenital malformations (3), but the evaluation and diagnosis of focal abdominal cystic lesions in infants can be divided into three categories based on their organ origin (4): solid organs (e.g., liver, kidneys, pancreas, ovary, spleen and adrenal glands), mesentery (e.g., mesenteric cyst, lymphatic malformation and cystic teratoma) or hollow organs (e.g., gastrointestinal duplication cysts in the bowel). Except for a few types of cysts, most abdominal cysts do not compromise the survival of the fetus, but approximately one-third of patients require surgical intervention (5). Surgical intervention should be reserved for cysts that are large at the outset or those that cause abdominal pain, abdominal distension, feeding difficulties, emesis, or organ damage in infants (5, 6). Thus, timely detection is crucial to optimize the management strategy *in utero* and to facilitate treatment in infancy, which reduces unnecessary interventions during pregnancy or avoids organ damage in infants.

Ultrasound is the preferred imaging modality for prenatal screening and diagnosis. With improvements in the resolution of ultrasound, regular ultrasound screening during pregnancy will identify more pregnancies with fetal intra-abdominal cysts (7). Magnetic resonance imaging is less frequently used but can be helpful in cases of difficult fetal cystic lesions when uncertainty remains after careful ultrasound evaluation (8, 9). However, accurate identification is challenging, even during childhood, due to the resemblance and diversity of the focal abdominal cystic lesions (9, 10). Accumulating evidence indicates that the pre- and postnatal outcomes of fetal abdominal cysts may be associated with organ origin, gestational age at diagnosis, ultrasound characteristics and size at diagnosis (7, 11–13). A recent multi-institutional retrospective cohort study reported that clear organ of origin, diagnosis earlier in gestation, initial prenatal cyst diameter and sonographic cyst characteristic changes can contribute to the differentiation and management of congenital ovarian cysts (14). However, a predictive model combining clinical characteristics and ultrasound features is still missing for relatively large groups, which may ultimately dictate the pre- and postnatal outcomes (e.g., surgical intervention, conservative management and spontaneous regression) of fetal intra-abdominal cystic lesions.

The ultrasound findings of fetal abdominal cystic lesions are usually non-specific, and the most common presentation is a round anechoic structure of varying size and cystic wall morphology. The aim of this study was to investigate the natural course of fetal abdominal cystic lesions from diagnosis *in utero* to postnatal outcome, to evaluate the diagnostic ability of prenatal ultrasound, and to develop a predictive model to determine spontaneous resolution and surgical intervention. Our findings may allow better prenatal counseling and management decisions for fetal abdominal cystic lesions.

## Materials and methods

### Study population

**Figure 1:**
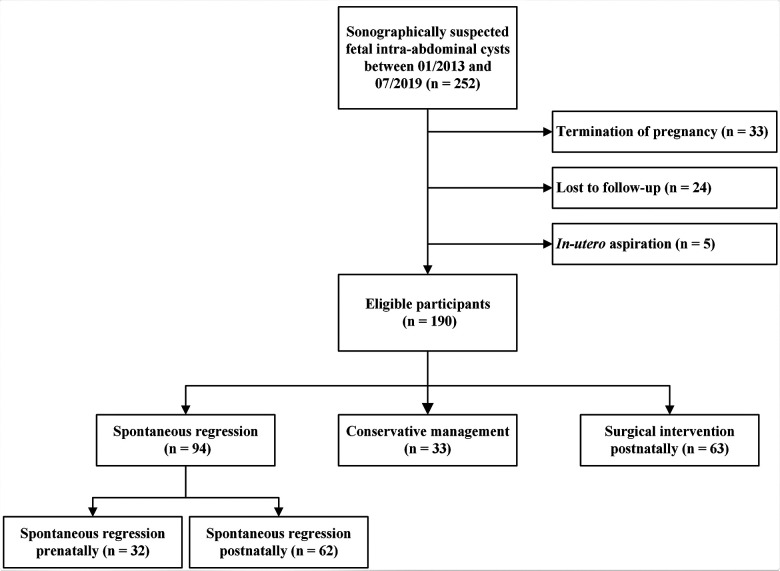
A trial flowchart summarizing fetal outcomes in the prenatal and postpartum cohorts.

In this longitudinal study, we retrospectively recruited fetuses according to the criteria proposed in three previous studies (4, 14, 15) from our group that were sonographically suspected as fetal intra-abdominal cysts at the Women’s Hospital, Zhejiang University School of Medicine, between January 2013 and July 2020. The exclusion criteria were as follows: (1) missing prenatal sonographic data files, (2) termination of pregnancy, and (3) *in utero* aspiration. Although gastrointestinal obstructions/atresia and urinary tract pathologies can present as cysts, they are typically tubular fluid-filled structures that can present in different ways (16). The postnatal outcomes were collected for a maximum of one year, or until surgical intervention or spontaneous regression. Figure 1 illustrates the enrollment of the participants in this study. The study was approved by the Institutional Review Board of the Women’s Hospital, Zhejiang University School of Medicine (approval number: IRB-20200231-R). All patients included in this study were anonymized and ascertained.

According to the Ranganath classification (4), the prenatal cystic lesions were classified as cysts of solid organs (originating from ovaries, kidneys, adrenal glands, gallbladder and spleen), cysts of hollow organs (originating from gastrointestinal duplication), peritoneal/mesenteric cysts, or non-specific cystic lesions (cystic lesions with unclear prenatal ultrasound diagnosis).

### Ultrasound parameters

A longitudinal series of prenatal sonographic files for each eligible patient was retrieved from the Picture Archiving and Communication System (Zhejiang Greenlander I.T. Co., Ltd., Hangzhou, China). All of the files were reviewed by two senior physicians with ultrasound experience, and difficult cases were verified by a third ultrasound diagnostic expert. The following characteristics of the intra-abdominal cysts were recorded according to previous reports (13, 14): (1) at the initial detection of the cyst: gestational age, initial cyst diameter, morphology (regular/irregular), components (cystic/cystic-solid), separation, cyst wall thickness, intra-cystic hemorrhage, localization and vascularity; (2) at gestational age of the ultrasound-diagnosed spontaneous regression; and (3) at 1 week before delivery: cyst diameter in persistent cases. Gestational age was determined by the last menstrual period and first-trimester ultrasound measurement of the crown-rump-length. Cyst wall thickening was defined as a cyst wall ≥0.2 cm in thickness. Based on the anatomical structure in humans, the localization of the abdominal cyst was classified as right upper abdomen, left upper abdomen, right lower abdomen, left lower abdomen or more than two quadrants.

### Clinical characteristics and follow-up

The maternal–fetal clinical data were retrieved from the Women’s Hospital, Zhejiang University School of Medicine, Hospital Medical Record Database (Shanghai Unionnet Co., Ltd., Shanghai, China) and included maternal age, obstetric history, number of fetuses, gestational age at delivery, mode of delivery (cesarean delivery or vaginal delivery), gender, birth weight and Apgar score.

At the 1-year follow-up, the postnatal outcomes were obtained from the Children’s Hospital, Zhejiang University School of Medicine, Hospital Electronic Medical Record System (Ewell Co., Ltd., Hangzhou, China). For the cases with no records at the Children’s Hospital, we interviewed mothers *via* telephone. The pre- or postnatal outcomes of the intra-abdominal cysts were classified as spontaneous regression, conservative management or surgical intervention, as previously reported (5, 17). Spontaneous regression was defined as spontaneous remission, which was usually monitored by ultrasound pre- or postnatally without any treatment (18). Surgical intervention included but was not limited to laparoscopy, laparotomy and transabdominal aspiration. For the cases that were treated by surgery, we recorded the reason of the intervention, organ origin, organ damage and pathological findings. Conservative management included cases with persistent intra-abdominal cysts that were observed without the need of surgical intervention.

### Statistical analysis

Data were statistically analyzed with SPSS 21.0 software (IBM SPSS Statistics for Windows, Armonk, NY, USA). Continuous data were tested for normality using the Kolmogorov–Smirnov test. The Student’s *t*-test was used for comparisons of normally distributed data, and the Mann–Whitney *U*-test was used for non-normally distributed continuous data.

In the predictive model of prenatal spontaneous regression, the clinical and ultrasound parameters contained gestational age at the first ultrasound examination, cyst diameter at the first ultrasound examination, morphology, components, separation, cyst wall thickness, detectable Doppler signal inside the cyst, intra-cystic hemorrhage and localization. The predictive model of postnatal surgical intervention was fitted to include the diameter before delivery in addition to the parameters described above. Univariate and forward stepwise multivariate logistic regression analyses were performed to determine the predictive factors for antenatal and postnatal outcomes. Receiver operating characteristic (ROC) curves were used to test the discriminative potential of the significant factors identified by logistic regression analysis. An area under curve (AUC) with a lower limit of 95% CI >0.5 was considered to be significant discrimination. The *Z*-test was used for pairwise comparisons of ROC curves. All the statistical tests were two-sided, and the data were considered statistically significant at *p *< 0.05.

## Results

### Maternal–neonatal clinical characteristics and pre- or postnatal outcomes

One-hundred ninety fetuses with intra-abdominal cysts were enrolled in our study, and maternal and neonatal clinical characteristics are summarized in Table 1. The Apgar scores at 1 and 5 min after birth were reportedly normal in all of the newborns. The male-to-female ratio was 1:2.82. The first- and second-trimester serum tests did not reveal evidence of chromosomal aneuploidy in all of the cases.

**Table 1:** Maternal and neonatal clinical characteristics.

Characteristics	Outcomes			
	Total (*n* = 190)	Spontaneous regression (*n* = 94)	Conservative management (*n* = 33)	Surgical intervention (*n* = 63)
Maternal age, year	29 (21–41)	29 (20–40)	29 (21–41)	29 (19–39)
**Obstetric history**				
Nulliparous	60 (31.58)	33 (35.11)	6 (18.18)	21 (33.33)
Multiparous	130 (68.42)	61 (64.89)	27 (81.82)	42 (66.67)
**Number of fetuses**				
Singleton	186 (97.89)	91 (96.81)	32 (96.97)	63 (100.00)
Twin pregnancy	4 (2.11)	3 (3.19)	1 (3.03)	0 (0.00)
GA at delivery, week	39 (28–42)	39 (33–42)	39 (34–40)	39 (28–41)
**Mode of delivery**				
Cesarean delivery	89 (46.84)	41 (43.62)	16 (48.48)	32 (50.79)
Vaginal delivery	101 (53.16)	53 (56.38)	17 (51.52)	31 (49.21)
Birth weight, g	3360 (1400–4650)	3355 (1450–4650)	3370 (2180–4460)	3360 (1400–4500)
**Gender**				
Male	51 (26.84)	21 (22.34)	10 (30.30)	20 (31.75)
Female	139 (73.16)	73 (77.66)	23 (69.70)	43 (68.25)
Apgar score at 1 min	10 (9–10)	10 (9–10)	10 (9–10)	10 (9–10)
Apgar score at 5 min	10 (10–10)	10 (10–10)	10 (10–10)	10 (10–10)

Continuous data are given as median (min-max) and categorical data as *n* (%).

GA, gestational age.

In brief, there were 49.47% (94/190) spontaneous regression cases (32 cases regressing prenatally and 62 cases regressing postnatally), 17.37% (33/190) conservative management cases and 33.16% (63/190) surgical intervention cases. Two cases died within one year of birth, diagnosed with cystic biliary atresia and adrenal neuroblastoma, respectively. The remaining cases (98.95%, 188/190) survived to the end of the follow-up period. The reasons for surgery included acute abdominal pain, feeding difficulties, intra-cystic hemorrhage, organ damage or functional insufficiency, large persistent cysts and compression of the surrounding organs. Moreover, 4 out of 14 ovarian masses were found to associate with intra-cystic hemorrhage or cyst wall thickening, including 3 cases complicated by torsion. For these 3 cases, the diameter before delivery on prenatal ultrasound was 6.6 cm, 4.6 cm, 5.2 cm, respectively.

### Diagnostic accuracy of prenatal ultrasound for the cases with surgical intervention

For the 63 cases who underwent surgical intervention, the histopathological findings and diagnostic prenatal ultrasound features are presented in Supplementary Table S1. There were nine cases of cystic lesions with unclear ultrasound diagnoses, and the rate of non-specific cystic lesions was 14.39% (9/63). For the 63 cases, prenatal ultrasound was found to identify fetal intra-abdominal cysts with 80.00% sensitivity (95% CI: 67.03%–89.57%), 37.50% specificity (95% CI: 8.52%–75.51%), 89.80% positive predictive value (95% CI: 83.51%–93.86%), 21.43% negative predictive value (95% CI: 8.80%–43.53%) and 74.60% accuracy (95% CI: 62.06%–84.73%), respectively. Moreover, the diagnostic accuracy of the cysts of solid organs, cysts of hollow organs and peritoneal/mesenteric cysts is shown in Table 2.

**Table 2:** Diagnostic accuracy of prenatal ultrasound records in 63 cases of intra-abdominal cysts with surgical intervention.

Prenatal ultrasound classification	Sensitivity		Specificity		PPV		NPV		Accuracy	
	%	95% CI	%	95% CI	%	95% CI	%	95% CI	%	95% CI
Cysts of solid organs	91.18	76.32–98.14	68.97	49.17–84.72	77.50	66.47–85.68	86.96	68.77–95.28	80.95	69.09–89.75
Cysts of hollow organs	75.00	42.81–94.51	100.00	93.02–100.00	100.00	100.00	94.44	86.45–97.84	95.24	86.71–99.01
Peritoneal/mesenteric cysts	50.00	15.70–84.30	96.36	87.47–99.56	66.67	30.29–90.20	92.98	86.87–96.37	90.48	80.41–96.42
All intra-abdominal cysts	80.00	67.03–89.57	37.50	8.52–75.51	89.80	83.51–93.86	21.43	8.80–43.53	74.60	62.06–84.73

PPV, positive predictive value; NPV, negative predictive value.

### Predictive factors for prenatal spontaneous regression

**Figure 2:**
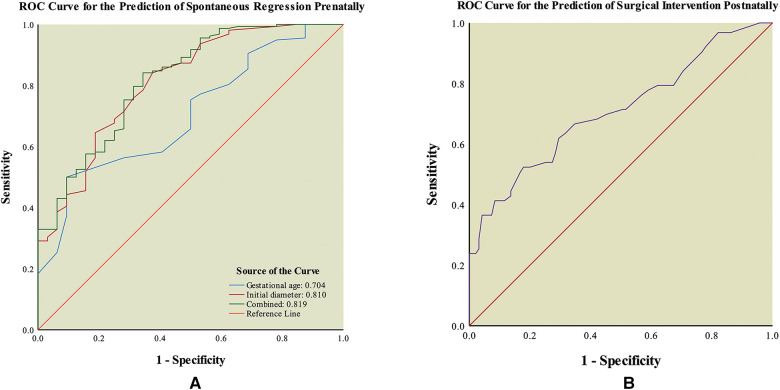
Receiver operating characteristic (ROC) curve of outcomes of fetal intra-abdominal cystic lesions. (**A**) ROC curve for the prediction of spontaneous regression prenatally. (**B**) ROC curve for the prediction of surgical intervention postnatally.

In univariate and multivariate logistic regression analyses, gestational age (Regression prenatally: 28.50 (13–37) week vs. Persistent postnatally: 32.50 (18–40) week) and initial diameter (Regression prenatally: 1.25 (0.50–3.40) cm vs. Persistent postnatally: 2.40 (0.80–11.50) cm) of the intra-abdominal cyst at the first ultrasound examination were identified as the independent predictors of prenatal spontaneous regression (both *p* < 0.05, Table 3). In ROC curve analysis, the AUC of gestational age was 0.704 (95% CI: 0.613–0.794), and the AUC of initial diameter was 0.810 (95% CI: 0.728–0.893), indicating that both parameters had discriminatory potential (Figure 2A). The other ultrasound parameters did not reach statistical significance.

**Table 3:** Univariate analysis and multivariate analysis of possible predictors of spontaneous regression prenatally in 190 fetuses.

Parameters	Outcomes		Univariate analysis		Multivariate analysis	
	Regression prenatally (*n* = 32)	Persistent postnatally (*n* = 158)	*p* value	OR (95% CI)	*p* value	OR (95% CI)
GA at the first ultrasound detection, week	28.50 (13–37)	32.50 (18–40)	<0.001	1.14 (1.07–1.21)	0.04	1.09 (1.01–1.19)
The diameter at the first ultrasound detection, cm	1.25 (0.50–3.40)	2.40 (0.80–11.50)	<0.001	4.04 (2.16–7.56)	<0.001	5.05 (2.33–10.94)
Morphology^a^			0.23	3.49 (0.45–27.33)	0.19	4.44 (0.48–41.53)
Regular	31 (96.88)	142 (89.87)				
Irregular	1 (3.12)	16 (10.13)				
Components			0.99	NA	0.99	NA
Cystic	32 (100)	147 (93.04)				
Cystic-solid	0 (0)	11 (6.96)				
Separation^a^			0.22	2.56 (0.57–11.43)	0.42	2.21 (0.33–14.92)
Yes	2 (6.25)	23 (14.56)				
No	30 (93.75)	135 (85.44)				
Thickness of cyst wall^a^			0.22	2.56 (0.57–11.43)	0.89	0.89 (0.16–4.88)
≥0.2 cm	2 (6.25)	23 (14.56)				
<0.2 cm	30 (93.75)	135 (85.44)				
Detectable Doppler signal inside the cyst			0.99	NA	0.99	NA
Yes	0 (0)	8 (5.06)				
No	32 (100)	150 (94.94)				
Intra-cystic hemorrhage			0.99	NA	0.99	NA
Yes	0 (0)	8 (5.06)				
No	32 (100)	150 (94.94)				
Localization^a^			0.47	1.11 (0.83–1.50)	0.08	1.95 (0.38–10.01)
Right upper abdomen	8 (25.00)	35 (22.15)				
Left upper abdomen	3 (9.38)	22 (13.93)				
Right lower abdomen	12 (37.50)	42 (26.58)				
Left lower abdomen	8 (25.00)	42 (26.58)				
More than two quadrants	1 (3.12)	17 (10.76)				

Continuous data are given as median (min-max) and categorical data as *n* (%). *p* value, odds ratio, and 95% confidence interval of the odds ratio were calculated using univariate/multivariate logistic regression analysis, comparing data between the group of spontaneous regression prenatally or not.

NA, not applicable; GA, gestational age.

^a^ OR value was specified to reference group (for categorical covariates).

The optimal threshold of gestational age was 32.5 weeks at the highest Youden’s index (0.41), with a sensitivity of 50.00% and specificity of 90.63%. Gestational age at ultrasound-diagnosis was found to be a significant predictor, with the possibility of prenatal spontaneous regression increasing by about 1.08-fold for every 1 week decrease in gestational age. The cut-off value of the initial diameter was 1.45 cm at the highest Youden index (0.47), with a sensitivity of 84.17% and specificity of 62.50%. The odds of prenatal spontaneous regression increased by 3.50-fold for every 1 cm decrease in diameter.

In addition, gestational age and initial diameter were integrated into the predictive model as y = −3.291 + 0.083 × gestational age + 1.252 × initial diameter. The model had an AUC value of 0.819 (95% CI: 0.739–0.899) for the prediction of prenatal spontaneous regression (Figure 2A). The optimal cut-off value was 0.74 at the highest Youden’s index (0.50), with a sensitivity of 84.18% and specificity of 65.63%. For the fetal intra-abdominal cysts, the lower the gestational age at diagnosis and the smaller the initial diameter, the greater the likelihood of prenatal spontaneous regression. Comparisons of the predictive accuracy showed no statistical difference between gestational age at diagnosis and initial diameter (*p* = 0.09, *z* = 1.70), between gestational age at diagnosis and the predictive model (*p* = 0.06, *z* = 1.87), and between initial diameter and the predictive model (*p* = 0.88, *z* = 0.15). These findings indicate that using the initial diameter alone was the most convenient method with a certain power for predicting prenatal spontaneous regression.

### Predictive factors for postnatal surgical intervention

For the 158 cases with intra-abdominal cysts at birth, univariate and multivariate logistic regression analyses revealed that the cyst diameter before delivery was significantly larger in the surgical intervention group [3.50 (1.00–11.00) cm] than that in conservative management and spontaneous regression groups [2.20 (0.50–4.80) cm] (*p* < 0.001, Table 4).

**Table 4:** Univariate analysis and multivariate analysis of possible predictors of surgical intervention postnatally in 158 fetuses with intra-abdominal cysts.

Parameters	Outcomes		Univariate analysis		Multivariate analysis	
	Spontaneous regression / Conservative management (*n* = 95)	Surgical interventio*n* (*n* = 63)	*p* value	OR (95% CI)	*p* value	OR (95% CI)
GA at the first ultrasound detection, week	33 (18–40)	32 (18–39)	0.20	1.04 (0.98–1.10)	0.14	1.05 (0.98–1.13)
The diameter at the first ultrasound detection, cm	2.30 (0.90–5.50)	2.80 (0.80–11.50)	<0.01	0.70 (0.55–0.88)	0.35	1.27 (0.77–2.11)
The diameter before delivery, cm	2.20 (0.50–4.80)	3.50 (1.00–11.00)	<0.001	0.55 (0.42–0.71)	<0.001	0.38 (0.23–0.63)
Morphology^a^			0.84	1.12 (0.39–3.25)	0.95	0.96 (0.28–3.35)
Regular	85 (89.47)	57 (90.48)				
Irregular	10 (10.53)	6 (9.52)				
Components^a^			0.10	0.25 (0.05–1.33)	0.42	0.44 (0.06–3.15)
Cystic	93 (97.89)	58 (92.06)				
Cystic-solid	2 (2.11)	5 (7.94)				
Separation^a^			0.94	1.04 (0.42–2.57)	0.27	1.90 (0.61–5.89)
Yes	14 (12.74)	9 (14.29)				
No	81 (85.26)	54 (85.71)				
Thickness of cyst wall^a^			0.20	0.56 (0.23–1.35)	0.12	0.42 (0.14–1.25)
≥0.2 cm	11 (11.58)	12 (19.05)				
<0.2 cm	84 (88.42)	51 (80.95)				
Detectable Doppler signal inside the cyst^a^			0.55	0.65 (0.17–2.69)	0.86	1.22 (0.14–10.76)
Yes	4 (4.21)	4 (6.35)				
No	91 (95.79)	59 (93.65)				
Intra-cystic hemorrhage^a^			0.70	0.78 (0.23–2.68)	0.99	1.01 (0.21–4.75)
Yes	6 (6.32)	5 (7.94)				
No	89 (93.68)	58 (92.06)				
Localization^a^			0.68	0.95 (0.74–1.21)	0.12	1.30 (0.94–1.80)
Right upper abdomen	22 (23.16)	13 (20.63)				
Left upper abdomen	14 (14.74)	8 (12.70)				
Right lower abdomen	22 (23.16)	20 (31.75)				
Left lower abdomen	29 (30.52)	13 (20.63)				
More than two quadrants	8 (8.42)	9 (14.29)				

Continuous data are given as median (min-max) and categorical data as *n* (%). *p* value, odds ratio, and 95% confidence interval of the odds ratio were calculated using univariate/multivariate logistic regression analysis, comparing data between the group of spontaneous regression prenatally or not.

GA, gestational age.

^a^ OR value was specified to reference group (for categorical covariates).

This model had an AUC of 0.710 (95% CI: 0.625–0.794) in ROC curve analysis, indicating that it had discriminatory potential for surgical intervention (Figure 2B). Its optimal cut-off value was 3.35 cm at the highest Youden’s index (0.35), with a sensitivity of 52.38% and specificity of 82.11%. The odds ratio was 0.548 (95% CI, 0.423–0.711).

## Discussion

Prenatal ultrasound screening is a valuable technique for the assessment of intra-abdominal cystic lesions; however, prenatal ultrasound may not provide an exact diagnosis. Most cystic lesions are managed non-operatively and associated with good perinatal outcomes, while some cysts must be removed. Based on the predictive model of pre- or postanal outcomes, this study showed that low gestational age at diagnosis and small initial diameter portend prenatal spontaneous regression, while diameter before delivery exhibits predictive power for the cases requiring postnatal surgical intervention. Thus, gestational age and the prenatal ultrasound characteristics are expected to predict the natural course and to guide the clinical management of cysts.

The prenatal diagnosis and postnatal management of intra-abdominal cystic lesions have important clinical implications. However, fetal intra-abdominal cystic lesions usually present with differential diagnoses, and their etiopathogenesis, histology and clinical presentation differ significantly (4, 15). Hyett et al. (1) indicated that most congenital lesions in the literature comprise relatively small case series, and thus, it is often difficult to arrive at a conclusive prenatal diagnosis. Hugele et al. (8) reported that the diagnostic accuracy of fetal intra-abdominal cysts by prenatal ultrasound was 51%. Recent results from the literature reported that magnetic resonance imaging (MRI) is a useful adjunct to prenatal ultrasound in the positioning of fetal abdominal cystic lesions, and could aid in characterization of the lesion in relationship to surrounding anatomic structures (3, 19). However, its value was limited and its diagnostic accuracy was 73.4% (8). It also remains to be investigated whether fetal MRI can help predict pre- and postnatal outcomes (14). In our study, intra-abdominal cysts with unclear prenatal ultrasound diagnoses were associated with lower diagnostic accuracy, with a sensitivity of 80.00%, specificity of 37.50%, positive predictive value of 89.80%, negative predictive value of 21.43% and accuracy of 74.60%, respectively. Therefore, precise diagnosis and etiological analysis have remained difficult, indicating that abdominal cystic lesions should be carefully monitored during the postnatal period.

The prognosis of most cases in this study was excellent, and only two infants died due to disease progression (biliary cirrhosis and adrenal neuroblastoma metastasis). Moreover, three cases with ovarian masses were complicated by torsion. In the present study, 49.47% of cases exhibited spontaneous regression and 33.16% of cases required surgical intervention. Overall, these results were consistent with those of Ozyuncu et al. (9), who reported that most cases did not require surgical intervention after birth. However, infants often require intensive treatment after birth and prolonged hospitalizations (20, 21). Therefore, a reliable method for the prediction of postnatal prognosis is crucial for minimizing the likelihood of irreversible consequences.

The prenatal evaluation of fetal intra-abdominal cystic lesions is dependent on ultrasound features such as the identification of solid components, thickened cyst walls with enhanced echo and large cyst size (10, 22). Previous research has indicated that gestational age at prenatal diagnosis, infant gender, anatomic location of the cyst, and largest documented size of the cyst are reliable predictors of postanal outcomes (23). Additionally, a systematic review has demonstrated that fetal abdominal cysts at 11–14 weeks of gestation are usually associated with good perinatal outcomes (7). Lewis et al. (12) reported that the cut-off value of the ovarian cyst diameter for the prediction of postnatal spontaneous regression was 3.75 cm. In our study, a system capable of stratifying fetal outcomes according to the clinical characteristics and ultrasound parameters was developed. The optimal threshold for predicting prenatal spontaneous regression was 32.5 weeks of gestation. As expected, the earlier the abdominal cystic lesions are detected, the greater the likelihood the lesions undergo prenatal spontaneous regression (17). In addition, the odds of achieving prenatal spontaneous regression increased by 3.50-fold for every 1 cm decrease in diameter. This study confirms that initial diameter is one of the key indices of spontaneous regression during pregnancy (12). We identified, for the first time, that diameter before delivery is an independent predictor of postnatal surgical intervention, and the best cut-off value was 3.35 cm. Noia et al. (24) showed that early aspiration of fetal ovarian cysts that exceed 4.0 cm in diameter associate with a good longitudinal prognosis, while Sanna et al. observed that 33% of cases increased antenatally in size (5). As observed by us and as previously reported by others, these dynamic observations may provide valuable information for the prediction of clinical outcomes in this population (25, 26). The predictive model affirms the need to combine ultrasound characteristics and gestational age at diagnosis for intra-abdominal cystic lesions to effectively predict fetal outcomes, and may be useful for improving future clinical management strategies.

### Limitations

The most important limitation of the present study is that participants exhibited diverse congenital abdominal cystic lesions and displayed a certain degree of non-specificity and diversity during the fetal period. Although the pre- or postnatal outcomes included surgical intervention, conservative management, and spontaneous regression, we did not discriminate between pathologies of fetal intra-abdominal cysts. This potentially represents a limitation of the analysis. Regardless, we adopted the criteria proposed in previous studies (4, 14, 15). However, there may still be some potential for overfitting in the predictive model, which may have impacted the reliability of the results. Moreover, this study was retrospective and nonrandomized in nature and based on a single-center cohort with a follow-up period of only one year. Further studies using larger sample sizes with longer follow-up data are required to validate our findings.

## Conclusion

In conclusion, we established a method based on prenatal ultrasound measurements and clinical characteristics to predict the prognosis of fetuses with intra-abdominal cysts. Despite the favorable long-term prognosis for most of the cases, it is important that patients requiring surgery receive accurate diagnoses and preventive interventions to preserve organ function. Future studies now need to verify this prediction model for clinical management in a larger prospective cohort study.

## Data Availability

The raw data supporting the conclusions of this article will be made available by the authors, without undue reservation.
