# Supplementary material for: Outcomes of sonographically-suspected fetal intra-abdominal cysts: Surgical intervention, conservative management and spontaneous regression
Source: Front Pediatr. 2022 Oct 18;10:1015678. doi: 10.3389/fped.2022.1015678 (PMC9891233; doi:10.3389/fped.2022.1015678)
Supplement: Supplementary file 1 [file Table1.docx]

**Supplementary Table 1. Comparison of prenatal ultrasound records and histopathological diagnosis in 63 cases with surgical intervention**

| **Prenatal ultrasound classification** | **Prenatal ultrasound diagnosis** | **Postanal histopathological diagnosis** |
| --- | --- | --- |
| Cysts of solid organs (n=40) | Ectopic kidney (n=4) | Ectopic kidney (n=4) |
|  | Kidney tumor (n=1) | Parasitic twin (n=1) |
|  | Adrenal cyst (n=2) | Adrenal simple cyst (n=1)  Adrenal neuroblastoma (n=1) |
|  | Ovarian cyst (n=17) | Intestinal duplication (n=1)  Mesenteric cyst (n=1)  Ovarian simple cyst (n=8)  Ovarian serous cystadenoma (n=1)  Peritoneal lymphangioma (n=1)  Urachal cyst (n=2)  Ovarian teratoma (n=3) |
|  | Ovarian teratoma (n=2) | Ovarian teratoma (n=2) |
|  | Choledochal cyst (n=13) | Hepatic teratoma (n=1)  Cystic biliary atresia (n=1)  Choledochal cyst (n=11) |
|  | Splenic cyst (n=1) | Splenic simple cyst (n=1) |
| Cysts of hollow organs  (n=9) | Gastric duplication (n=6) | Gastric duplication (n=6) |
|  | Intestinal duplication (n=3) | Intestinal duplication (n=3) |
| Peritoneal/mesenteric cysts (n=6) | Peritoneal lymphangioma (n=4) | Peritoneal lymphangioma (n=3)  Intestinal duplication (n=1) |
|  | Peritoneal teratoma (n=2) | Retroperitoneal teratoma (n=1)  Peritoneal teratoma (n=1) |
| Non-specific cystic lesions (n=9) | undefined diagnosis by prenatal ultrasound (n=9) | Urachal cyst (n=1)  Omphalocele with cyst formation (n=1)  Giant hepatic cyst (n=1)  Intestinal duplication (n=1)  Imperforate hymen with hydrocolpos (n=1)  Ovarian simple cyst (n=1)  Peritoneal lymphangioma (n=2)  Choledochal cyst (n=1) |
